# Energy consumption features, correlative factors, and management strategies of tertiary hospitals across various climate zones in Jiangsu, China

**DOI:** 10.3389/fpubh.2026.1750668

**Published:** 2026-03-04

**Authors:** Xiaolin Ni, Yu Pan, Lixin Peng, Xintong Li, Haibo Xu

**Affiliations:** 1 School of Management, Xuzhou Medical University, Xuzhou, China; 2 Department of Logistics Management, The Affiliated Hospital of Xuzhou Medical University, Xuzhou, China; 3 Medical Insurance Department, The Affiliated Hospital of Xuzhou Medical University, Xuzhou, China; 4 Medical Affairs Department, The Affiliated Hospital of Xuzhou Medical University, Xuzhou, China

**Keywords:** correlative factors, energy conservation, energy consumption, public health, tertiary hospitals

## Abstract

**Introduction:**

In line with China’s “Dual Carbon” goals, achieving a green transformation of the healthcare system is imperative. This study aims to identify the factors driving energy use and to explore energy-saving strategies in large tertiary general hospitals across various architectural climate zones, thereby providing insights for sustainable development within the healthcare sector.

**Methods:**

Adopting a research design of “quantitative as the main approach, supplemented by qualitative” methods, we integrated data analysis, field surveys, and questionnaires. The study examined 12 tertiary general hospitals in Jiangsu Province from 2022 to 2023, collecting on-site building data and energy management information. Data analysis combined Spearman correlation analysis with findings from the questionnaire surveys.

**Results:**

The analysis revealed that hospitals in different climate zones exhibited similar quarterly patterns of energy use, but the key correlative factors varied significantly. Specifically, energy consumption in cold zones was primarily affected by operational scale, whereas in hot-summer-cold-winter zones, it was influenced by both hospital size and external temperature. Field surveys indicated that although all sampled hospitals had implemented some energy-saving technologies and management systems, none had installed real-time online energy monitoring systems.

**Conclusion:**

To establish efficient, low-carbon, and resilient hospitals, tailored strategies are recommended. For cold zones, the focus should be on optimizing energy structures, building design, and facility layout. For hot-summer-cold-winter zones, improving building insulation is crucial. Furthermore, accelerating investment in intelligent carbon-monitoring systems, managing energy hardware upgrades at the source, and enhancing staff energy-saving awareness are essential steps forward.

## 1 Introduction

The current global society faces an unprecedented climate crisis. Climate warming not only threatens ecosystem stability but also directly endangers human health through pathways such as heat stress, infectious disease transmission, air pollution, and extreme weather events, which significantly increase mortality and disease burdens, posing severe challenges to public health security (1–3). As the largest developing country, driven by its inherent needs for sustainable development and its responsibility to build a global community of shared future, China announced the “Dual Carbon” goals (peaking carbon emissions by 2030 and achieving carbon neutrality by 2060) in September 2020. These goals, a crucial part of China’s ecological civilization construction, are not only key initiatives for promoting the nation’s green transition but are also regarded as an important public health strategy with significant health co-benefits (4).

Energy conservation in public institutions is vital for achieving the “Dual Carbon” goals. The global health community is speeding up the transition of healthcare systems toward a model that balances low-carbon practices, resilience, and equity, aiming to deliver high-quality services without increasing environmental impacts or risking the health of future generations (5). According to the “*China Urban–Rural Construction Carbon Emissions Research Report (2024)*,” (65) building energy use accounts for over 20% of the country’s total consumption and continues to grow. Among these, government agencies, hospitals, and schools make up nearly 90% of total energy consumption in public institutions. Hospitals, as crucial sites for safeguarding public health, operate continuously at high intensity, leading to energy use that can be 1.6 to 2.0 times that of typical public buildings (6). The carbon emissions during their operation mainly come from the energy needed to maintain medical environments and run equipment (7). This high-carbon-emission profile makes hospitals a key focus for emission-reduction efforts within the health sector. Therefore, promoting energy conservation and carbon reduction in hospitals is not only about conserving resources but also about improving healthcare quality and strengthening the resilience of public health institutions (8, 9). In recent years, research has increasingly emphasized the direct link between hospital carbon emissions and public health (10–12). As health service providers, hospitals’ carbon emissions indirectly influence population health by intensifying climate change, while coordinated energy conservation and reduction efforts can bring substantial public health benefits (13, 14).

**Figure 1:**
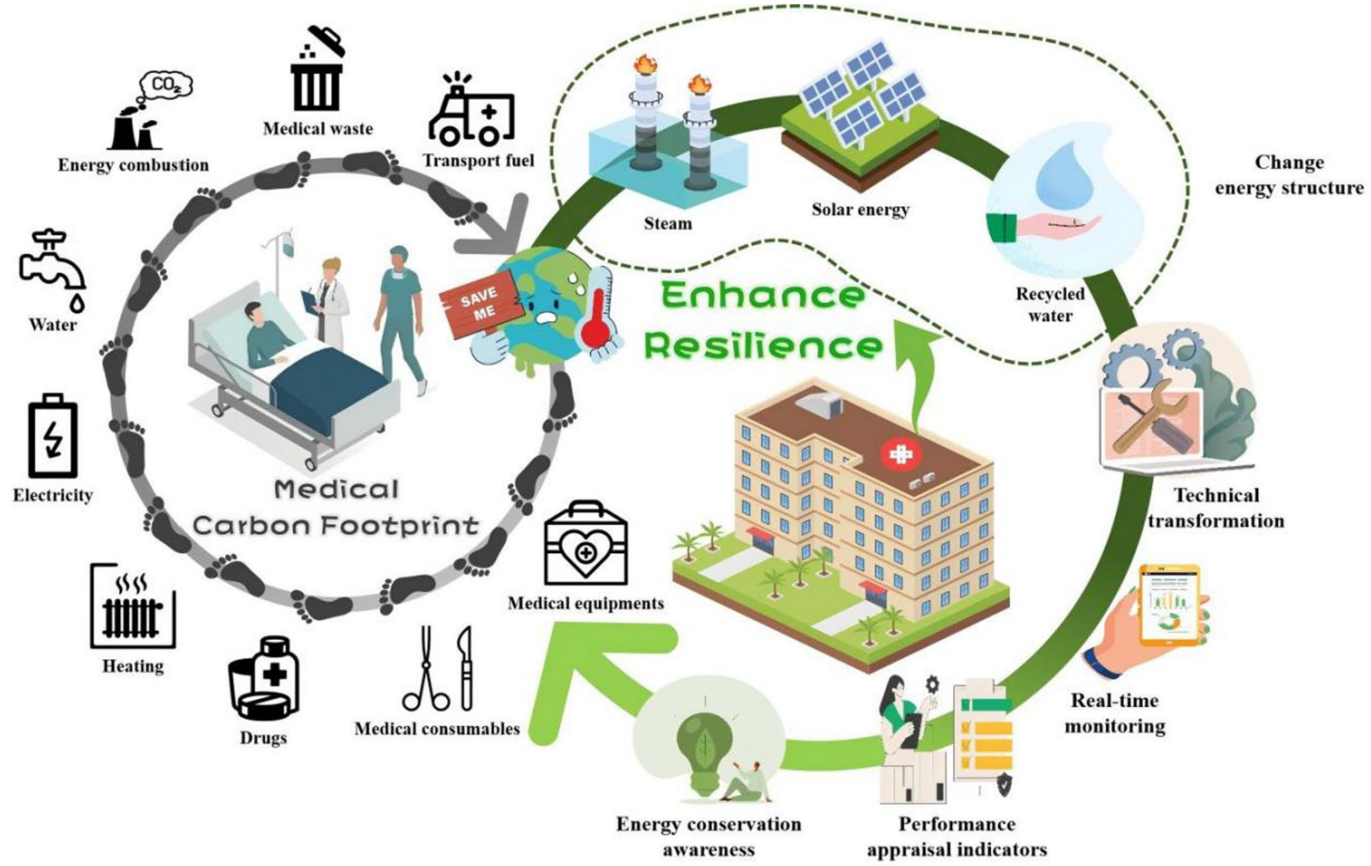
Schematic diagram of carbon footprint and mitigation strategies in healthcare institutions. The left cycle shows sources of carbon emissions during operational phases, while the right cycle displays mitigation measures. The dashed box highlights a group of strategies related to changing the energy mix. Illustrations from WPS Office.

As high-energy-consuming public buildings, hospitals produce significant carbon emissions through their operations, posing a major challenge at the crossroads of environmental sustainability and public health resilience. A complex range of factors influences this carbon footprint (Figure 1). Regarding the energy structure, distinct differences exist in the energy mix and consumption intensity among hospitals located in different climatic zones. Research indicates that cold regions, due to higher heating demands, generally exhibit higher energy consumption and carbon emissions than warmer regions (15). For example, in Cyprus, hospitals in coastal and inland areas rely on electricity for 60% of their energy needs, whereas those in mountainous regions depend on oil for 80% of their heating requirements (16). Regarding influencing factors, numerous studies have employed correlation analysis to identify associations between hospital energy consumption and operational indicators (17, 18), though the specific factors vary by region. Furthermore, several studies have applied regression analysis to confirm the impact of various variables on hospitals’ overall energy consumption (19–22). Optimizing energy management is a context-specific process that demands customized strategies tailored to local climate and socioeconomic conditions to identify the best low-carbon technological options (23). Effective strategies include increasing staff awareness, implementing incentive policies, adopting technological innovations, and thoroughly overhauling energy management systems to achieve energy savings, reduce emissions, and maintain healthcare quality (24, 25). Data-driven methods combining energy audits, structured surveys, and on-site inspections with both quantitative and qualitative models are essential for developing targeted improvements and behavioral interventions (25, 26).

China’s “Dual Carbon” goals offer a strong policy framework for the healthcare sector, promoting connections between emission reduction, public health, and systemic resilience (27, 28). As the largest and most resource-intensive medical institutions, tertiary general hospitals are a crucial leverage point for widespread change. Creating low-carbon, resilient hospital energy systems is essential for ensuring operations continue during extreme climate events and reflects the sector’s social responsibility and health mission. China’s vast landmass, diverse climates, and varied energy systems result in significant regional and seasonal differences in hospital energy use (29). The findings further reflect that climatic conditions and heating/cooling source types are key factors influencing carbon emissions from building energy consumption (30–32). Studies show that operational factors such as building size, bed numbers, and service volume are strongly associated with energy use, but the primary influence varies by region (33–35). According to recent studies conducted in several provinces of China, general hospitals typically consume more energy than specialized hospitals (36), and larger hospitals have greater potential for energy savings and low-carbon management (37). Nonetheless, developing effective, targeted decarbonization strategies depends on a deeper understanding of what drives hospital carbon emissions across different climate zones, an area where research remains limited.

To address this gap, this study conducts an empirical analysis of 12 tertiary general hospitals in Jiangsu Province, China. Jiangsu, a socio-economically advanced province (ranked second in GDP) with a population of over 85 million and a unique transect spanning both cold-summer and hot-summer cold-winter climate zones, provides an ideal setting for comparative cross-climatic research. By combining data analysis, field investigations, and questionnaire surveys, this study aims to: (1) characterize the carbon emission profiles of the hospitals; (2) identify the main factors driving carbon emissions in the two distinct climate zones; and (3) develop differentiated, climate-adaptive energy management strategies that consider public health resilience. The novelty of this research lies in two key contributions: First, it goes beyond single-region analyses by pioneering a comparison of hospitals across cold and hot-summer-cold-winter zones, highlighting how climate adaptability influences energy management. Second, it constructs a multidimensional analytical framework that integrates medical service data, building attributes, and climate variables, addressing a gap in the literature between operational factors and physical building conditions. The findings aim to provide evidence-based policy guidance for the sustainable, low-carbon, and resilient development of tertiary hospitals in China. They may serve as a model for healthcare systems in similar climatic zones worldwide.

## 2 Materials and methods

### 2.1 Research design

This study adopted a research methodology that prioritizes quantitative analysis with qualitative support. Data collection involved gathering operational and energy consumption data from 12 healthcare institutions for statistical analysis. Field surveys were conducted to gain an intuitive understanding of the basic building information and energy-use patterns of the sample institutions, aiding triangulation with the database. A questionnaire survey was to collect the objective facts systematically, so as to supplement the field survey data. The questionnaire was developed based on the framework proposed by Aunión-Villa et al. (38), which covers key aspects of hospital decarbonization, including energy audits, energy-efficient equipment retrofits, energy management systems, and the use of renewable energy. The instrument was further refined, referencing relevant items from the “*Guidelines for Energy Audits of Office Buildings of State Organs and Large Public Buildings*” (Appendix B) as a structured data collection tool. The target respondents were energy management officers at the 12 hospitals. A purposive sampling method ensured that one senior manager with comprehensive knowledge completed the questionnaire at each institution. The survey was conducted offline. Trained investigators visited the hospitals to conduct face-to-face interviews and collected data on three main areas: basic building information, profiles of energy-using equipment, and energy conservation practices. All questionnaire data collected were thoroughly checked at the time of collection to ensure accuracy and reliability.

### 2.2 Data standardization

This study selected Jiangsu Province—a region spanning different architectural climate zones in China (Appendix A)—as the research area. A total of 12 tertiary general hospitals, including six from a cold climate zone and six from a hot-summer-cold-winter climate zone, were included as data sources. Medical operational data on energy consumption were extracted as variables and standardized following the *National Performance Evaluation Manual for Tertiary Public Hospitals (2024)* issued by China’s National Health Commission (66). The standardized names, abbreviations, and units of all variables used in this study are provided in Table 1. The conversion coefficients for various energy consumption types, expressed in tonnes of standard coal equivalent, are summarized in Table 2.

**Table 1:** Standardized terminology, abbreviations, and measurement units.

Measurement variables	Abbreviations	Unit
Building areas	BA	m^2^
Number of buildings	NB	building
Available beds	AB	bed
Outpatient visits	OPV	person
Inpatient admissions	ADM	person
Surgical volumes	SV	person
Average length of stay	ALOS	day
Revenue	REV	10^4^ CNY
Temperature	T	°C
Energy consumption	EC	tce
Energy consumption per revenue	ECR	CNY
Comprehensive energy consumption per unit area	CECUA	kgec/(m^2^ × a)

The calculation formula of the variables is as follows.

(1) Average length of stay = Total bed days occupied by discharged patients/inpatient admissions.

(2) Energy consumption per revenue = Total energy consumption expenditure/total medical revenue.

(3) Comprehensive energy consumption per unit area = Annual total energy consumption/building areas.

**Table 2:** Calculate energy consumption in tons of standard coal equivalent.

Energy forms	Pricing unit	Equivalent to the ton standard coal coefficient (tce)
Water	t	0.0857 × 10^−3^
Electricity	kW·h	0.1229 × 10^−3^
Steam	MJ	0.0341 × 10^−3^
Natural gas	Nm^3^	1.33 × 10^−3^

Source from operational manual for performance appraisal of national tertiary public hospitals (2024).

### 2.3 Survey database

This study used two primary data sources. The first dataset came from the *Jiangsu Tertiary Healthcare Institutions Medical Quality and Operational Performance Bulletin*, published by the Jiangsu Medical Administration Service Guidance Center. Quarterly medical operational data for the 12 sampled hospitals during 2022–2023 were collected, including outpatient visits, inpatient admissions, surgical volumes, average length of stay, and revenue figures. The second dataset was obtained from hospital energy management departments and their contracted third-party energy management service companies. This dataset included monthly actual consumption records and billing documents for all utility types during 2022–2023. To reconcile the temporal granularity between the monthly energy data and the quarterly operational data, we performed rigorous temporal alignment during database construction. Using natural quarters (Q1: January–March, Q2: April–June, etc.) as the alignment benchmark, monthly energy consumption data within each quarter are aggregated into “quarterly total energy consumption” and matched with the corresponding medical operation data. The sum of the four quarters is then calculated as “annual total energy consumption.”

### 2.4 Statistical analysis

Data organization and database construction were performed using Microsoft Excel 2010, and statistical analyses were conducted with SPSS 25.0. For the medical institutional operational and energy consumption data, characteristics were summarized using descriptive statistics, including frequency distributions, trend analyses, and tables. The normality of all variables was assessed with the Shapiro–Wilk test. Based on the results, independent-samples t-tests were used for normally distributed data to compare groups, while the nonparametric Mann–Whitney U test was applied for non-normally distributed data (39, 40). To identify correlative factors of hospital energy consumption, correlation analyses were conducted: Pearson’s correlation for normally distributed variables and Spearman’s correlation for non-normally distributed variables (41). A two-sided *p*-value of less than 0.05 was considered statistically significant.

## 3 Results

### 3.1 Basic information of tertiary general hospitals in different architectural climate zones

The 12 tertiary general hospitals sampled in Jiangsu Province had an average floor area of 214,534 m^2^ and an average of about 9 buildings per campus. The average number of open beds was 1,821. The average quarterly consumption of water, electricity, steam, natural gas, and total energy equivalent was 11.20, 671.84, 452.21, 130.88, and 1,266.13 tce, respectively. Detailed energy consumption data for each hospital are shown in Table 3.

**Table 3:** Energy consumption of tertiary general hospitals in various architectural climate zones.

Climate zones	Samples	Annual energy consumption (2022/2023)(tce/year)				ECR (CNY)
		Water	Electricity	Steam	Natural gas	
Cold zones	1	88.4/94.9	4,877.5/5,055.0	8,111.4/7,786.2	94.5/102.7	158.8/124.9
	2	150.3/170.6	7,095.3/6,970.8	6,202.7/6,201.1	0.00/0.00	225.3/215.94
	3	30.6/33.0	2,869.9/2,878.5	0.00/0.00	2,114.2/2,000.0	231.61/184.3
	4	17.3/17.9	1,225.0/1,118.1	0.00/0.00	0.00/0.00	102.5/91.2
	5	22.4/19.3	1,047.7/1,008.79	0.00/0.00	706.9/634.0	174.5/148.4
	6	19.8/22.0	2,076.3/2,086.9	0.00/0.00	0.00/0.00	215.9/206.7
Hot and cold zones	7	31.2/37.5	2,175.7/2,212.1	1,387.9/1,540.1	568.5/564.5	170.1/179.9
	8	30.5/34.4	2,075.5/2,048.9	2,239.0/2,092.6	0.00/0.00	78.0/64.4
	9	32.1/34.9	1,707.7/1,752.2	1,523.0/1,448.4	0.00/0.00	192.2/159.7
	10	29.8/29.8	2,473.7/2,646.3	2,292.7/2,587.1	0.00/0.00	210.0/220.2
	11	26.7/26.5	1,831.1/1,770.9	0.00/0.00	1,960.2/1,905.0	347.1/217.6
	12	39.2/35.9	2,713.4/2,779.0	0.00/0.00	961.5/952.6	234.8/180.5

### 3.2 Analysis of energy consumption characteristics of tertiary general hospitals across different building climate zones

**Figure 2:**
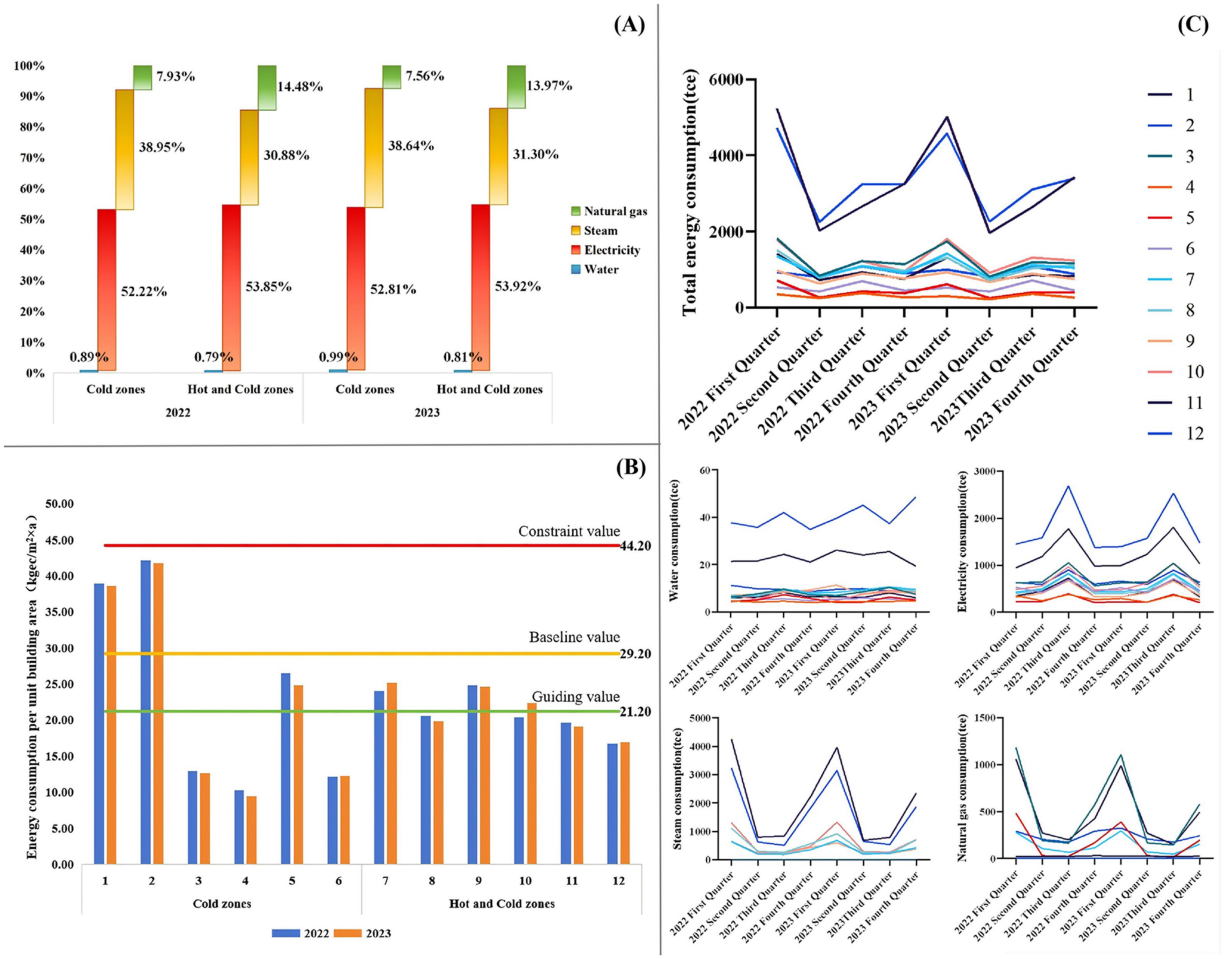
Analysis of energy consumption characteristics in tertiary general hospitals across different architectural climate zones: **(A)** Energy mix of tertiary general hospitals in various architectural climate zones (*n* = 12); **(B)** Comparison of energy consumption per unit floor area in tertiary general hospitals across different climate zones with the energy quota standards for public institutions in Jiangsu Province; **(C)** Quarterly energy consumption patterns of tertiary general hospitals in different climate zones.

Analysis of energy composition and proportional distribution (Figure 2A) and the Kruskal–Wallis test revealed that electricity was the dominant energy source in the sub-item energy consumption statistics of 12 tertiary general hospitals in Jiangsu Province for the 2022–2023 year, accounting for more than half of total consumption(*H* = 14.44, *p* = 0.002). Hospitals in cold-climate zones showed a greater dependence on steam than those in hot-summer-cold-winter zones, which had a relatively higher proportion of natural gas use.

To align with the national “Dual Carbon” goals for energy management and carbon reduction, Jiangsu Province adopted the local standard, *Energy Consumption Quotas and Calculation Methods for Public Institutions (DB32/T4001-2021)* (67). This standard sets limits, baselines, and guiding values for annual energy use per unit area in healthcare facilities, with maximums at 44.2, 29.2, and 21.2 kgce/(m^2^ × a), respectively. A comparison of actual energy use in sampled hospitals with these quotas (Figure 2B) showed that all 12 hospitals had energy consumption below the annual per-unit-area constraint limit. In cold-climate zones, two hospitals exceeded the baseline, one surpassed the guiding value, and three stayed below the guiding limit. In hot-summer-cold-winter zones, three hospitals went over the guiding value, while three remained below it.

Quarterly data on total and categorized energy consumption for the 12 hospitals from 2022 to 2023 (Figure 2C) showed a relatively consistent pattern of quarterly variation across climate zones. Total energy consumption was higher in the first and third quarters than in the second and fourth quarters, peaking in the first quarter and showing an annual “W”-shaped trend. Water consumption showed minimal quarterly fluctuation across all hospitals. Electricity consumption was higher in the second and third quarters, peaking in the third quarter with an “inverted V”-shaped trend. Steam consumption for Hospitals 1, 2, 7, 8, 9, and 10 was higher in the first and fourth quarters, following a “U”-shaped trend; the remaining six hospitals did not use steam. Regarding natural gas, Hospitals 2, 4, 6, 8, 9, and 10 did not use it for regular energy needs. In contrast, the other six hospitals showed higher consumption in the first and fourth quarters, also following a “U”-shaped trend.

### 3.3 Analysis of correlative factors for energy consumption in tertiary general hospitals across different architectural climate zones

The results of Mann–Whitney U tests indicated that the ECR was 173.32 ± 61.40 CNY in cold zones and 187.87 ± 88.42 CNY in hot-summer-cold-winter zones (U = 1,047, *p* = 0.442). The CECUA was 5.88 ± 12.20 kgce/m^2^ in cold zones and 5.30 ± 9.38 kgce/m^2^ in hot-summer-cold-winter zones (U = 1,153, *p* = 0.992). Total EC was 1519.80 ± 1455.15 tce in cold zones and 1012.46 ± 271.78 tce in hot-summer-cold-winter zones (U = 1,257, *p* = 0.442). These findings demonstrate that, under a unified provincial energy conservation and carbon reduction policy framework, no statistically significant differences in total energy consumption were observed between hospitals in different climate zones (Table 4). It represents the reduction in differences in energy consumption among hospitals across different building climate zones, driven by unified provincial policies.

**Table 4:** Mann–Whitney U test on energy consumption of tertiary general hospitals across different architectural climate zones.

Variables	Cold zones (M ± SD)	Hot and cold zones (M ± SD)	*U*	*p*
ECR	173.32 ± 61.40	187.87 ± 88.42	1,047	0.442
CECUA	5.88 ± 12.20	5.30 ± 9.38	1,153	0.992
Total EC	1,519.80 ± 1,455.15	1,012.46 ± 271.78	1,257	0.442
Water	14.30 ± 13.51	8.09 ± 1.51	1,322	0.213
Electricity	798.14 ± 606.72	545.55 ± 189.66	1,017	0.323
Steam	589.61 ± 1,116.74	314.81 ± 342.78	1,337	0.147
Natural gas	117.75 ± 261.16	144.01 ± 228.46	1,263	0.385

The data covered 8 quarters in 2022/2023 and included six tertiary general hospitals in each of the two architectural climate zones (*n* = 48).

Spearman correlation analysis revealed that in cold zones, total energy consumption exhibited the strongest positive correlation with the NB (*ρ* = 0.926, *p* < 0.01), followed by strong positive correlations with SV (*ρ* = 0.831, *p* < 0.001), AD (*ρ* = 0.768, *p* < 0.001), REV (*ρ* = 0.767, *p* < 0.001), BA (*ρ* = 0.717, *p* < 0.001), and AB (*ρ* = 0.795, *p* < 0.001). In contrast, no significant correlations were found with climatic variables (quarterly average high or low temperatures). In hot-summer-cold-winter zones, the correlation patterns were distinctly different and generally weaker. Total energy consumption showed only weak to moderate positive correlations with the AB (*ρ* = 0.380, *p* < 0.01), BA (*ρ* = 0.333, *p* < 0.05), and AD (*ρ* = 0.308, *p* < 0.05). A weak negative correlation was observed with the quarterly average low temperature (*ρ* = −0.332, *p* < 0.05). No statistically significant associations were found with other medical service volume indicators, including OPV, SV, and REV (Table 5).

**Table 5:** Spearman correlation analysis of correlative factors for total energy consumption in tertiary general hospitals across different building climate zones.

Architectural climate zones	Variables	Total EC	
		*ρ*	*p*
Cold zones	BA	0.717^***^	< 0.001
	NB	0.926^**^	< 0.01
	AB	0.795^***^	< 0.001
	OPV	0.603^***^	< 0.001
	AD	0.768^***^	< 0.001
	SV	0.831^***^	< 0.001
	ALOS	0.061	0.912
	REV	0.767^***^	< 0.001
	Quarterly average high temperature	−0.128	0.386
	Quarterly average low temperature	−0.129	0.384
Hot and cold zones	BA	0.333^*^	< 0.05
	NB	0.146	0.322
	AB	0.380^**^	< 0.01
	OPV	0.039	0.791
	AD	0.308^*^	< 0.05
	SV	0.250	0.078
	ALOS	0.095	0.521
	REV	0.212	0.148
	Quarterly average high temperature	−0.278	0.056
	Quarterly average low temperature	−0.332^*^	< 0.05

*p < 0.05; **p < 0.01; ***p < 0.001.

| *ρ* | > 0.7 indicates strong correlation, 0.4 < | *ρ* | ≤ 0.7 indicates moderate correlation, and | *ρ* | ≤ 0.4 indicates weak correlation.

**Figure 3:**
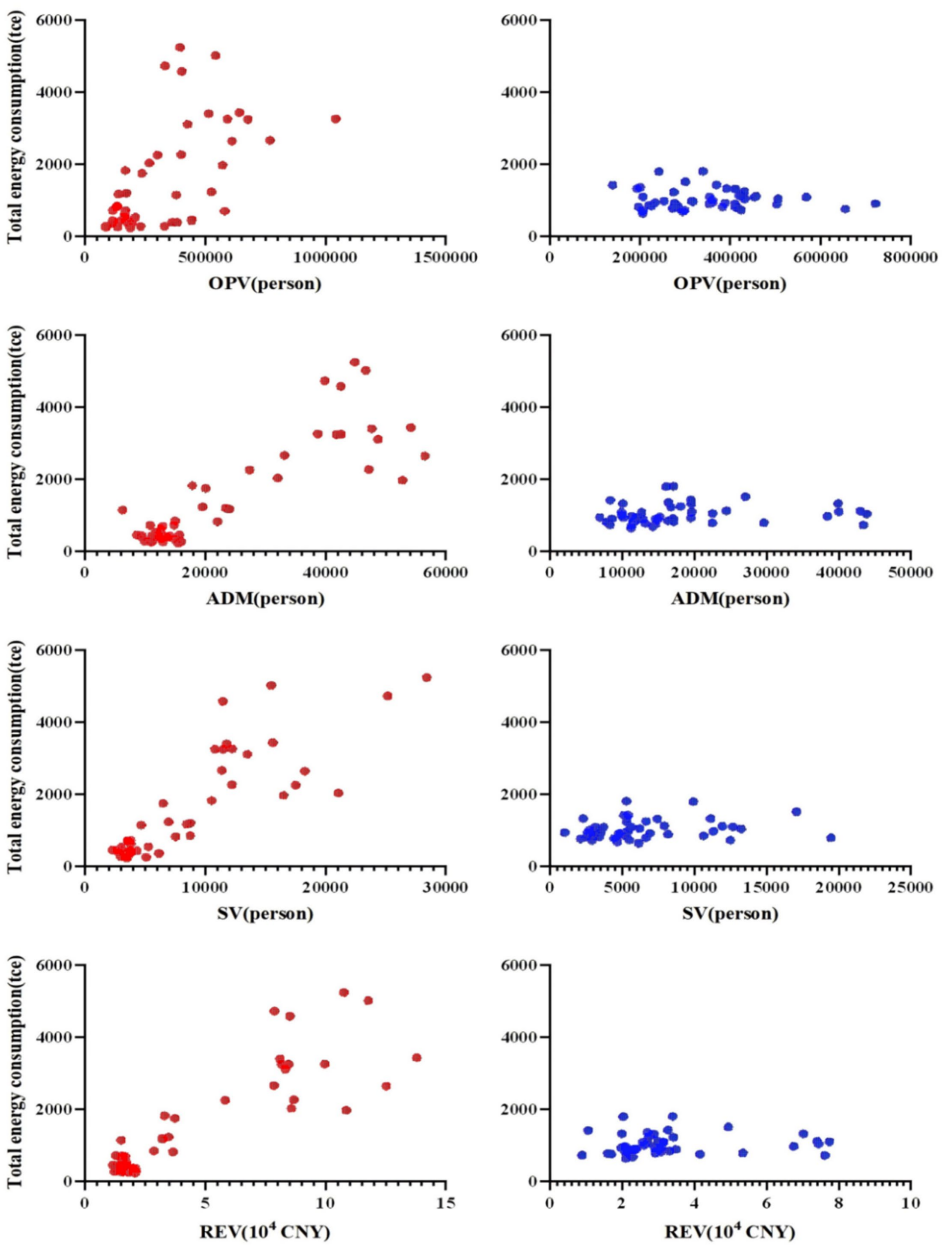
Scatter plot of correlative factors for total energy consumption in tertiary general hospitals across different building climate zones. (1) Red dots represent data from cold climate zones; blue dots represent data from hot-summer-cold-winter zones. (2) Each data point represents the quarterly data from an individual hospital.

Scatter plots illustrating the relationship between quarter total energy consumption (vertical axis) and four key correlative factors—outpatient visits, inpatient admissions, surgical volumes, and revenue (horizontal axis)—are presented in Figure 3. In cold-climate zones, data points showed high dispersion, with total energy consumption increasing as operational scale or revenue increased. Spearman correlation analysis confirmed strong positive correlations between total energy consumption and surgical volume (*ρ* = 0.831, *p* < 0.001), patient discharges (*ρ* = 0.768, *p* < 0.001), and revenue (*ρ* = 0.767, *p* < 0.001). This study is the first to incorporate the number of buildings as a potential correlate of energy consumption. Results indicated that, in addition to building areas and available beds, energy consumption in cold-climate hospitals showed a robust positive correlation with the number of building complexes (*ρ* = 0.926, *p* < 0.01). In contrast, hospitals located in the hot-summer-cold-winter climate zones showed weaker correlations between total energy consumption and medical service volumes (e.g., inpatient admissions: *ρ* = 0.308, *p* = 0.033). As shown in Figure 3, data points in these zones were more clustered, primarily concentrated in the lower-left quadrant, indicating a general trend toward lower energy consumption and no clear linear relationship with outpatient visits, inpatient admissions, surgical volumes, or revenue.

### 3.4 Survey on energy conservation management in tertiary general hospitals across different building climate zones

Survey results (Table 6) showed that all 12 hospitals had adopted energy-saving technology upgrades and set up energy management systems, including equipment replacement, lighting upgrades, pipeline maintenance, and sub-metering. Notably, five hospitals had implemented renewable energy systems—mainly photovoltaic power, air-source and ground-source heat pumps, solar water heating, and rainwater harvesting. However, none of the hospitals had real-time energy monitoring systems. Hospitals in hot-summer-cold-winter climate zones performed better in energy audits and in renewable energy use than those in cold-climate zones. Additionally, only 25% of hospitals provided systematic energy conservation training, while more than half lacked dedicated energy management positions or regular energy consumption analysis.

**Table 6:** Statistics from the questionnaire survey on energy conservation and carbon reduction measures in tertiary general hospitals

Architectural climate zones	Cold zones						Hot and cold zones					
Hospital samples	1	2	3	4	5	6	7	8	9	10	11	12
Whether there is a sub-item measurement of energy consumption.	√	-	√	√	-	√	√	√	√	-	√	√
Whether to use renewable energy.	-	-	-	√	-	√	-	-	√	√	√	-
Whether an energy audit has been carried out.	-	-	-	-	√	√	-	-	√	√	√	√
Whether energy-efficient technological retrofitting has been carried out.	√	√	√	√	√	√	√	√	√	√	√	√
Whether to install an online energy consumption monitoring system.	-	-	-	-	-	-	-	-	-	-	-	-
Whether to carry out regular training on energy conservation.	-	-	√	-	-	-	-	√	√	-	-	-
Whether to establish an energy conservation management system.	√	√	√	√	√	√	√	√	√	√	√	√
Whether to conduct regular energy consumption analysis.	√	-	-	√	-	√	-	√	-	-	√	-
Whether to establish energy management positions.	√	-	-	√	-	√	-	-	√	√	√	-

‘√’ means ‘yes’; ‘-’ means ‘no’.

## 4 Discussion

This study shows that electricity is the primary energy source for tertiary general hospitals in Jiangsu Province, with notable regional differences: cold zones have higher steam demand. In contrast, hot-summer-cold-winter zones rely more on natural gas. These results highlight significant opportunities to optimize energy systems to improve climate resilience in healthcare facilities. Additionally, the primary factors driving energy use vary by climate zone, requiring region-specific energy management strategies that suit local conditions while ensuring the continuous delivery of essential medical services. Energy-saving efforts remain inconsistent across hospitals, with apparent gaps in real-time monitoring and in systematic conservation training. Going forward, focus should be on promoting data-driven, precision energy management and establishing comprehensive carbon-monitoring systems. These actions are vital to advancing high-quality, sustainable development in the healthcare sector.

### 4.1 Optimizing energy systems in healthcare facilities to improve climate resilience

Based on the findings above, the total energy consumption of the 12 sampled healthcare institutions in Jiangsu Province peaked in the first and third quarters, displaying a transparent “W”-shaped pattern. Space heating was the primary driver of the winter peak, while cooling-related electricity use contributed to the secondary summer peak, consistent with previous studies on hospital energy use (31, 42, 43). In the cold climate zone, two hospitals had energy consumption per unit floor area significantly above the regional average, making them key targets for decarbonization. Specifically, Samples 1 and 2 had notably higher winter energy use due to steam-based heating systems. Additionally, their expansion and renovations—done without integrated energy-efficient design — created a complex mix of centralized, split, and VRV HVAC systems across multiple buildings, resulting in high overall and per-square-foot energy use. In contrast, Sample 3, also in the cold zones and similar in size, had much lower energy consumption because it used natural gas for heating. Similarly, Samples 4 and 6, which utilized electricity-driven renewable systems (ground-source and air-source heat pumps), showed no significant difference in total energy use compared to Sample 5 (which used natural gas), despite their larger building areas. These findings highlight the better performance of renewable systems in reducing emissions. In regions with hot summers and cold winters, differences in total energy use among hospitals were less pronounced, likely because of similar building sizes.

Energy optimization can be positioned as an enabling indicator to enhance climate resilience and adaptation capacity of public health systems (44–48). The diverse energy profiles across Jiangsu’s climate zones highlight the need to shift from basic energy conservation to resilient, tailored carbon management strategies. (1) Since electricity is the primary energy source, developing distributed energy resources with storage and smart microgrids is essential. This ensures a continuous power supply to critical areas like emergency and ICU departments, while also boosting energy independence and climate resilience (49, 50). (2) Cold zones in Jiangsu Province, heavy reliance on steam for centralized heating requires upgrading older systems and incorporating renewables to cut fossil fuel use (25, 51). Installing sub-meters for HVAC, lighting, and hot water systems is crucial for monitoring energy consumption and guiding targeted retrofits (52). (3) For the hot-summer-cold-winter zones, where natural gas is widely used for heating and hot water, priorities should include electrification (such as implementing air-source heat pumps) and enhancing building insulation. These region-specific strategies not only lower carbon emissions but also reduce operational costs, freeing up resources to improve medical services and equipment. Future work could further investigate improvements in energy management and the low-carbon transition to support operational continuity and reduce system vulnerability, including service availability during extreme events, power outage recovery time, and patient outcomes.

### 4.2 Improving energy efficiency in healthcare facilities through technological upgrades

In cold climate zones, the total energy consumption of healthcare institutions shows a significant upward trend as operational scale and revenue increase, demonstrating strong positive correlations with surgical volume (*ρ* = 0.831, *p* < 0.001), inpatient admissions (*ρ* = 0.768, *p* < 0.001), and revenue (*ρ* = 0.767, *p* < 0.001). These findings indicate that energy use in cold zones is primarily driven by medical service volume and facility size, supporting previous research (15, 19, 36, 53) and confirming that energy is a crucial input for delivering high-quality healthcare services. Therefore, energy conservation measures should be adopted without compromising medical quality or patient safety, especially in regional medical centers where higher energy consumption reflects their vital role in emergency care and complex procedures. This suggests that energy policies should prioritize structural optimization over simple consumption reduction. This study is the first to identify the number of buildings as a strong predictor of total energy use (*ρ* = 0.926, *p* < 0.01), indicating that incorporating energy-efficient design and low-carbon technologies during infrastructure development can reduce operational emissions at the source and improve energy efficiency. Such strategies help maintain access to and the fairness of essential healthcare services.

International evidence shows that low-carbon upgrades to electrical, heating, and cooling systems can recoup costs within a few years (54–56). These improvements provide net positive health benefits by improving indoor and outdoor air quality, thereby helping reduce chronic disease burdens among patients and healthcare workers and creating economic incentives for hospital energy retrofits (57). Therefore, when implementing energy-saving efforts, hospitals should schedule upgrades to high-energy equipment during low-demand periods (Q2 and Q4) to minimize disruptions to medical operations (58).

Given the challenge of standardizing energy consumption data across different climate zones, we strongly recommend that hospitals adopt intelligent energy systems to achieve precise energy conservation through data-driven approaches (41, 59–61). Installing intelligent monitoring and control systems allows real-time tracking of energy usage patterns and automatic adjustments of supply based on actual demand, preventing unnecessary waste. Special attention should be given to strengthening energy data analysis during peak consumption periods (Q1 and Q3), implementing targeted area monitoring, promoting behavioral saving strategies, and dynamically adjusting equipment operation parameters according to outdoor temperatures (62).

### 4.3 Enhancing green development of healthcare institutions via policy-driven energy conservation awareness

Establishing a low-energy, low-emission healthcare system represents a strategic “preventive” public health investment with long-term significance. Based on data analysis, this study finds that the provincial unified energy consumption management policy may have effectively mitigated the potential impact of climatic factors on hospital energy consumption. The current standardized target-oriented energy consumption control model demonstrates feasibility at the provincial level and can serve as a reference template for implementation in other regions. Policy leadership should go beyond simply “promoting energy-saving technologies.” It is crucial to incorporate refined “carbon intensity” metrics—such as carbon emissions per bed or per medical service unit—along with broader environmental performance indicators into the core evaluation systems for hospital accreditation, high-quality development assessments, and green hospital standards (63). Such institutional mechanisms can help shift hospital administrators’ mindset from a traditional “cost-control” approach to a “value-investment” perspective, encouraging them to prioritize the environmental health impact of hospital operations with the same rigor as healthcare quality. This change is essential for guiding the healthcare sector toward a sustainable development model that equally emphasizes climate resilience and health outcomes.

Conversely, survey results showed that only one-quarter of hospitals conducted systematic energy conservation training. Prior research suggests that increasing organization-wide awareness of energy savings can be more effective than simply buying new equipment or making structural changes (22, 64), and a combined approach of “technological upgrades and policy guidance” can successfully turn this awareness into concrete actions (23). Therefore, hospital leadership should prioritize carbon reduction by implementing ongoing training and awareness campaigns for administrators, clinical staff, and patients. Initiatives like gathering energy-saving suggestions and hosting conservation competitions can encourage active employee involvement and help build a culture focused on overall energy conservation.

### 4.4 Limitation

Although this study thoroughly examined the energy consumption characteristics and correlative factors of tertiary hospitals across different climate zones in Jiangsu Province, China, and proposed context-specific management strategies, the following limitations remain. First, despite grouping by climate zone, the energy consumption data from the 12 tertiary general hospitals were not adjusted for climate normalization (e.g., degree days). Additionally, the small sample size and strong collinearity in healthcare operational data hindered the development of robust regression models for energy consumption. However, this limitation underscores the need for hospital energy management to increasingly adopt data-intelligent approaches, such as implementing strategies based on “degree days” to develop more realistic and actionable energy efficiency measures. Second, the sample was limited to tertiary general hospitals. Although this focus clarifies energy consumption patterns in extensive medical facilities, the lack of comparison with hospitals of other grades in the same climate zones may limit the general applicability of the proposed energy management and carbon reduction strategies. Third, we fully recognize the importance of controlling confounding variables to enhance research rigor. However, in this study, to maintain the homogeneity of the study sample, variables such as hospital size, service intensity, climatic zone, and case composition were not controlled. Finally, while Jiangsu Province includes different architectural climate zones, the climatic differences within this region are relatively minor. Future research could validate its broader applicability through cross-provincial and cross-level sample expansion, providing more targeted data support and policy recommendations for refined energy consumption management in medical institutions across different climate zones, administrative divisions, and hierarchical levels.

## 5 Conclusion

This study systematically analyzed 12 tertiary general hospitals across different architectural climate zones in Jiangsu Province to assess their energy consumption characteristics, correlative factors, and current energy conservation practices. The results showed that while the quarterly trends of total energy consumption were similar across climate zones, notable differences existed in energy structures and main correlative factors: energy use in cold climate zones was mainly driven by medical service volume and building physical parameters (such as the number of buildings), whereas in hot-summer-cold-winter zones, it was more closely related to climatic conditions. Field investigations further indicated that implementing renewable energy technologies effectively reduced the impact of operational growth on energy consumption. However, hospital-wide awareness of energy savings and intelligent management measures remains limited and needs improvement. At the policy level, we recommend incorporating “carbon emission intensity” into the core indicator system for hospital accreditation and high-quality development evaluation. This would shift the focus of hospital energy conservation and emissions reduction from merely controlling total energy consumption to strategic investments aimed at enhancing the public health system’s climate resilience and improving population health outcomes. Concerning energy-saving and carbon-reduction strategies, hospitals in cold zones should focus on optimizing energy mixes, building designs, and medical facility layouts to decrease unnecessary energy use. Conversely, those in hot-summer-cold-winter zones should prioritize improving building insulation for better thermal efficiency. The next phase of decarbonization efforts should prioritize source-level energy management through hardware upgrades, investment in intelligent carbon-monitoring systems with high-frequency sub-metering and data analytics capabilities, and the enhancement of staff energy-saving awareness. These measures are essential for fully unlocking the potential of existing technologies and personnel, enabling genuine performance-based management, and ultimately achieving the goals of improving healthcare service quality, reducing carbon emissions, and establishing green and sustainable hospitals.

## Data Availability

The original contributions presented in the study are included in the article/Supplementary material, further inquiries can be directed to the corresponding author.
