# Supplementary material for: Energy consumption features, correlative factors, and management strategies of tertiary hospitals across various climate zones in Jiangsu, China
Source: Front Public Health. 2026 Mar 4;14:1750668. doi: 10.3389/fpubh.2026.1750668 (PMC12997266; doi:10.3389/fpubh.2026.1750668)
Supplement: Supplementary file 1 [file Image_1.pdf]

frontiers

Appendix A

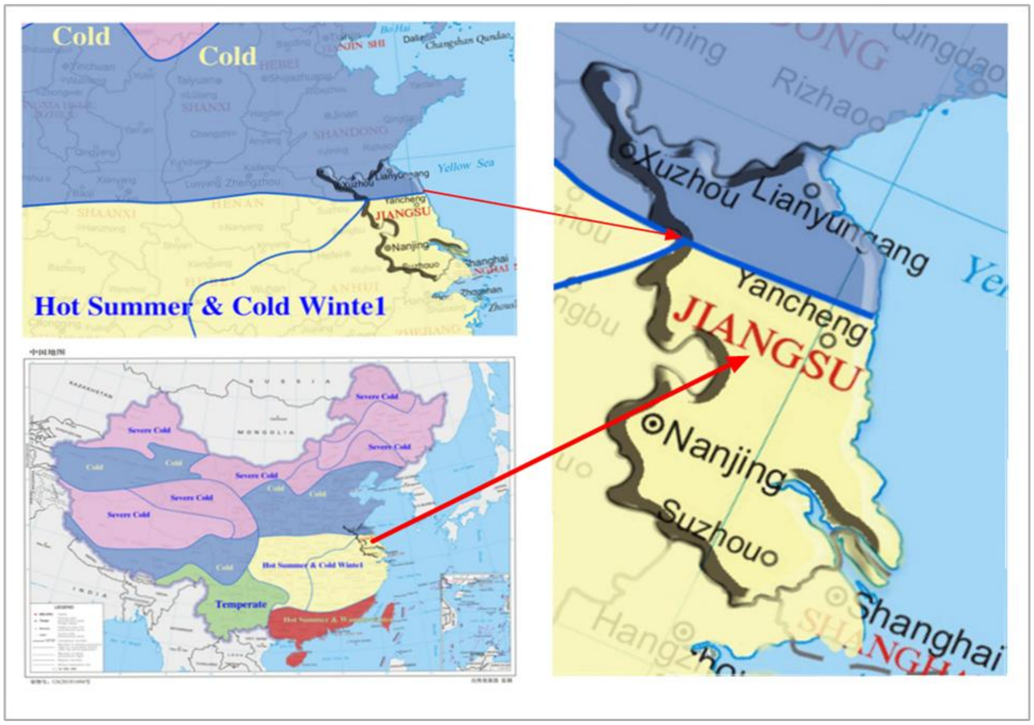

Figure A.1: Map of Jiangsu Province's Location Within China's Architectural Climate Zones

Note: The map of Jiangsu Province's administrative divisions is from the standard map service website (http://bzdt.ch.mnr.gov.cn/), and the climate zone map is from the Standard of Climatic Regionalization for Architecture (GB50178-93) (1993) *. The authors have made the necessary edits.

* National Bureau of Technical Supervision of the People's Republic of China, Ministry of Housing and Urban-Rural Development of the People's Republic of China (1993). Standard of Climatic Regionalization for Architecture (GB50178-93). China Planning Press: Beijing, China, 1993. (In Chinese)

Appendix B*

* Ministry of Housing and Urban-Rural Development of the People's Republic of China. Notice on Issuing the "Guidelines for Energy Audits of Office Buildings of State Organs and Large-scale Public Buildings" [Internet]. Beijing, China. October 31, 2007 [cited 2025 June 18]. Available from: https://www.mohurd.gov.cn/gongkai/zc/wjk/art/2007/art_17339_158568.html. (Article in Chinese)

Questionnaire on Energy Conservation and Carbon Reduction Strategies in Tertiary General Hospitals in Jiangsu Province

I Basic Building Information
1. Building Name
2. Number of Buildings
3. Building areas________m^2
4. Climate Zone

☐ Severe Cold ☐ Cold ☐ Hot Summer & Cold Winter
☐ Hot Summer & Warm Winter ☐ Temperate

II Energy Equipment Information
1. Cooling Equipment

☐ Water-cooled Chillers ☐ Air-source Heat Pumps
☐ Variable Refrigerant Flow (VRF) Systems ☐ Lithium Bromide Absorption Chillers
☐ Water-source Heat Pumps ☐ Water-loop Heat Pumps ☐ Air Conditioners
☐ Others (Please Specify: _____)

2. Heating Equipment

☐ Gas/Oil-fired Boilers ☐ Electric Boilers ☐ District Heating Network
☐ Air-source Heat Pumps ☐ Lithium Bromide Absorption Hot Water Units ☐ VRF Systems
☐ Air Conditioners ☐ Water-source Heat Pumps ☐ Cogeneration Units
☐ Solar Water Heating ☐ Others (Please Specify: _____)

3. Domestic Hot Water Equipment

☐ Gas/Oil-fired Boilers ☐ Electric Boilers ☐ Air-source Heat Pumps
☐ Solar Water Heaters ☐ Water-source Heat Pumps ☐ Cogeneration Units
☐ Externally Supplied Steam/Hot Water ☐ Others (Please Specify: _____)

4. Lighting Types

☐ Incandescent Lamps ☐ Standard Fluorescent Lamps ☐ Slim-tube Fluorescent Lamps
☐ Compact Fluorescent Lamps ☐ Sodium Lamps ☐ Mercury Lamps

III Energy Efficiency Management
1. Is Energy Consumption Sub-metered by System?

☐ Yes (Types of Sub-metering: ☐ Water ☐ Electricity ☐ Steam ☐ Natural Gas
☐ Others (Please Specify: _____)
☐ No

2. Is Renewable Energy Utilized?

☐ Yes (Types: ☐ Solar Water Heating ☐ Photovoltaic (PV) ☐ Ground-source Heat Pumps ☐ Air-source Heat Pumps ☐ Recycled Water ☐ Others (Please Specify: _____)
☐ No

3. Has an Energy Audit Been Conducted?

☐ Yes (Year: _____)
☐ No

4. Has Energy conservation Retrofitting Been Performed?

☐ Yes (Year: _____; Retrofitted Areas: _____)
☐ No

5. Is an Online Energy Monitoring System Installed?
☐ Yes
☐ No

6. Are Regular Energy Efficiency Training Programs Conducted?
☐ Yes (Frequency: _____)
☐ No

7. Has an Energy Management System Been Established?
☐ Yes (Year: _____)
☐ No

8. Is Periodic Energy Consumption Analysis Performed?
☐ Yes (Frequency: _____)
☐ No

9. Are Dedicated Energy Management Positions Established?
☐ Yes (Number of Staff: _____)
☐ No

Appendix C

Calculate energy consumption in tons of standard coal equivalent and carbon accounting emission

Energy forms	Pricing Unit	Equivalent to the ton standard coal coefficient (tce)	Carbon Accounting Emission	
			2022	2023
Water	t	0.0857×10^{-3}	0.168×10^{-3} tCO_2e/t [1]	0.168×10^{-3} tCO_2e/t [1]
Electricity	kW·h	0.1229×10^{-3}	0.5978×10^{-3} $tCO_2e/kW \cdot h$ [2]	0.5827×10^{-3} $tCO_2e/kW \cdot h$ [3]
Steam	MJ	0.0341×10^{-3}	0.11×10^{-3} tCO_2e/MJ [4]	0.11×10^{-3} tCO_2e/MJ [4]
Natural gas	Nm^3	1.33×10^{-3}	2.16×10^{-3} tCO_2e/Nm^3 [1]	2.16×10^{-3} tCO_2e/Nm^3 [1]

Note: Source from

[1] Jiangsu Province Civil Building Carbon Emission Calculation Guidelines.

[2] Announcement on the Release of 2022 Power Carbon Dioxide Emission Factors (Ministry of Ecology and Environment, National Bureau of Statistics)

[3] Announcement on the Release of 2023 Power Carbon Dioxide Emission Factors (Ministry of Ecology and Environment, National Bureau of Statistics)

[4] Greenhouse Gas Emission Accounting Method and Reporting Guidelines for Public Building Operating Enterprises (Trial)
